# Periatrial Epicardial Fat Is Associated with Markers of Endothelial Dysfunction in Patients with Atrial Fibrillation

**DOI:** 10.1371/journal.pone.0077167

**Published:** 2013-10-15

**Authors:** Nicolas Girerd, Alina Scridon, Francis Bessière, Samuel Chauveau, Alain Geloen, Loic Boussel, Elodie Morel, Philippe Chevalier

**Affiliations:** 1 Rhythmology Department, Louis Pradel Cardiology Hospital, Hospices Civils de Lyon, Bron, France; 2 Physiology Department, University of Medicine and Pharmacy of Targu Mures, Targu Mures, Romania; 3 Unité 870 INSERM / INRA 1235, INSA, La Doua, Villeurbanne, France; 4 Radiology Department, Croix-Rousse Hospital, Hospices Civils de Lyon, Lyon, France; 5 Lyon Reference Center for Inherited Arrhythmias, Louis Pradel Cardiovascular Hospital, Lyon, France; University of Illinois at Chicago, United States of America

## Abstract

**Background:**

Epicardial adipose tissue (EAT) is associated to atrial fibrillation (AF) burden and outcome after AF ablation. We intended to determine whether global or local EAT is associated with systemic and/or left atrial (LA) inflammation and markers of endothelial dysfunction in AF patients.

**Methods and Results:**

Total, atrial, and ventricular EAT volume (EATtotal, EATatrial, EATventricular) were measured by multislice cardiac CT in 49 patients with paroxysmal (PAF, n=25) or persistent AF (PeF, n=24). Periatrial epicardial fat thickness at the esophagus (LA-ESO) and thoracic aorta (LA-ThA) were also measured. Vascular endothelial growth factor (VEGF), interleukin-8 (IL-8), soluble intercellular adhesion molecule 1 (sICAM-1), transforming growth factor-β1 (TGF-β1), and von Willebrand Factor (vWF) levels were measured in peripheral and LA blood samples obtained during catheterization during AF ablation. Patients with PeF had higher EATatrial (*P*<0.05) and LA-ESO (*P*=0.04) than patients with PAF. VEGF, IL-8, and TGF-β1 were not associated with EAT. In contrast, after adjusting for LA volume and body mass index, higher LA-ThA was significantly associated with higher sICAM-1 and vWF levels, both in peripheral blood (*P*<0.05) and in LA (*P*<0.05). Similar results were found with LA-ESO. Body mass index, EATtotal and EATventricular were not associated with sICAM-1 and vWF.

**Conclusions:**

Periatrial epicardial fat showed a significant positive association with increased levels of sICAM-1 and vWF, which are biomarkers of endothelial dysfunction. No such associations were found when considering body mass index or EATtotal. These results suggest that local EAT rather than regional or total adiposity may modulate endothelial dysfunction in patients with AF.

## Introduction

Obesity, as measured by body mass index (BMI), is a well known risk factor for atrial fibrillation (AF) initiation [1] and maintenance [2]. Recently, much attention has been devoted to the link between body fat distribution and AF genesis. More specifically, there has been a focus on the role between epicardial fat [3–8], which is – the true visceral fat of the heart, and AF. Epicardial adipose tissue (EAT) volume is higher in patients with either paroxysmal AF (PAF) or persistent AF (PeAF) than in controls [3]. EAT is also associated with poor outcome after AF ablation [7,8], and a causal link between EAT and AF genesis has been suggested by animal studies [9,10]. In patients with PAF or PeAF, high dominant frequency sites are located near EAT sites [11], suggesting that EAT may modulate electrophysiological properties.

The mechanisms underlying the association between epicardial fat and AF remain unclear [4,8,12,13]. It has been suggested that epicardial fat produces pro-inflammatory cytokines, which in turn have a regional effect on cardiac myocytes [12,13]. The involvement of neurally- mediated mechanisms with vagal modulation of atrial electrophysiology via fat pads has also been hypothesized [11,12].

Obesity [14], and especially visceral obesity [15–17], has been identified as a key trigger of endothelial dysfunction. In sinus rhythm patients, abnormal EAT is associated with endothelial dysfunction [18]. To date, no study has addressed this association in AF patients. Recent data support the link of endothelial dysfunction with the pathophysiology of AF [19–21]. Moreover, endothelial dysfunction is associated with higher risk of stroke [22] and lower probability of recovery of sinus rhythm in this patient group [19,23]. These observations indicate that endothelial dysfunction might represent the main link between epicardial fat and poorer prognosis for AF patients.

In this study, we investigated the association of 1) EAT volume and 2) the distribution of EAT with systemic and intracardiac levels of several biomarkers of inflammation and/or endothelial dysfunction.

## Methods

The methodology used in this study was reported previously [24]. A total of 72 patients scheduled for AF catheter ablation at the Rhythmology Department of the Louis Pradel Cardiology Hospital in Lyon, France between August 2008 and June 2010, were included in the REMI study [24]. The 49 patients (PAF, n=25, PeF, n=24) who underwent cardiac CT angiography in our institution prior to the ablation procedure were enrolled in this substudy.

Atrial fibrillation was defined as ‘paroxysmal’ when the arrhythmia was self-terminating within 7 days and ‘persistent’ when the AF episode persisted for 7 days or pharmacological or electrical cardioversion were required to terminate the arrhythmia. Patients being treated with anti-inflammatory or steroid treatments were excluded.

The study protocol was approved by the South-East II People’s Protection Committee and by the Advisory Committee on Information Processing in Research in the Field of Health. All participants provided written informed consent.

### Blood Sampling and Laboratory Analysis

Blood sampling was performed during routine cardiac catheterization prior to any ablation procedure or administration of heparin. Blood samples were obtained from the femoral vein through one of the venous sheaths. The left atrium (LA) was accessed through transseptal puncture, and a blood sample was taken from the LA via the transseptal sheath. Serum concentrations of soluble intercellular adhesion molecule-1 (sICAM-1) and transforming growth factor- β1 (TGF- β1) were measured using commercially available quantitative enzyme-linked immunosorbent assay kits (Human TGF- β1 and Human sICAM-1/CD54Quantikine ELISA kits, R&D Systems, Inc., Minneapolis, MN). Luminex methodology was used to measure the serum levels of vascular endothelial growth factor (VEGF) and interleukin-8 (IL-8) with R&D System kits (Human VEGF and Human CXCL8/IL-8 Fluorokine Multi-analyte Profiling ELISA, R&D Systems, Inc.) The lower limits of detection were 4 pg/mL for VEGF, IL-8, and TGF- β1, and 30 ng/mL for sICAM-1. Serum concentrations of CRP were measured using a latex immunoassay with an automated clinical analyser (Abbott Architect, Abbott Laboratories, Rungis, France), which has a lower limit of detection of 0.2 mg/L. As recommended, CRP values higher than 10 mg/L were excluded from the analysis[25]. Plasma von Willebrand factor (vWF) was determined on a BCS analyzer (Siemens Healthcare Diagnostics, Inc., Deerfield, IL) with a Samsung Kit test (Marburg, Germany) by immunoturbidimetric assay using polystyrene particles coated with polyclonal rabbit antihuman vWF antibodies. The technicians performing the analysis were not aware of the patient’s characteristics or adiposity data.

### Imaging Protocol

Contrast-enhanced multidetector cardiac CT was performed using a Brilliance 64 scanner (Philips Healthcare, Best, the Netherlands) with iomeprol (Iomeron 400; Bracco Diagnostics, Milan, Italy) injected into the right cubital vein with an 18-gauge catheter. The patient was placed in a supine headfirst position. Acquisitions were performed on the entire heart area in the head-to-feet direction. The following parameters were used: 64 detectors, individual detector width of 0.625 mm, retrospective ECG gating, tube voltage of 120 kV, tube current of 300 mAs, pitch of 0.2, and half-rotation reconstruction. Eighty milliliters of iomeprol and then 50 mL of saline solution were injected at 3 mL/sec. A bolus-tracking method was used with an attenuation threshold of 180 Hounsfield units (HU) in the ascending aorta. Reconstruction parameters for the axial sections were a 1.5-mm effective section thickness, 1-mm increments, a reconstruction filter CB, and an adapted field of view. Retrospective ECG-gated reconstruction was performed at 75% of the R-R interval. To obtain adequate gating and minimal motion artifacts, subjects with heart rates >80 beats/min received beta-blockers.

### Image Analysis

Image reconstructions for EAT and LA volume measurement were performed at 75% of the R-R interval. EAT measurement was performed on 3-mm thick axial slices with no interslice gap. The reader was required to manually trace the pericardium. EAT was defined as any adipose tissue located within the pericardial sac. Total EAT volume (EATtotal), atrial EAT volume (EATatrial), and ventricular EAT volume (EATventricular) were measured using the freely available OsiriX imaging software (Foundation, Geneva, Switzerland). Fat voxels were identified using threshold attenuation values of -190 to -30 HU. We set the right pulmonary artery as the upper slice limit and the slice below the posterior descending coronary artery as the lower slice limit. We chose this lower boundary to better distinguish epicardial fat from pericardial fat that was localized around the diaphragm. Once the total EAT was measured, the images were resliced into 3-mm thick slices parallel to a plane constructed from the lateral portions of the tricuspid and mitral annulus. EATatrial and EATventricular were calculated from these resliced images using the same methodology used to calculate EATtotal.

The regional thickness of EAT was determined next. In a short-axis view, the periatrial epicardial fat thickness was measured as the shortest distance (in mm) between the mid-LA wall and 3 anatomic landmarks: esophagus (LA-ESO), main pulmonary artery (LA-PA), and descending thoracic aorta (LA-ThA). The slice that provided the shortest distance was used for each measurement. This methodology was used previously by others [4]. As described by Shin and colleagues[5], we also measured the right and left atrioventricular grooves (RAVG and LAVG), the interventricular groove (IVG), and the interatrial septum thickness (IAS) in a 4- or 5-chamber long-axis view. IAS was measured distantly from the initial portion of the aorta.

To determine the LA volume, the images were resliced into 5-mm thick slices parallel to the mitral annulus plane. The endocardial border of the LA was manually traced for each slice. The LA appendage and the pulmonary veins were excluded at their junction with the LA on each image.

Measurements were performed by a single investigator (N.G.), and the intraobserver reproducibility was 0.98 (intraclass correlation) for measurements of EATtotal and 0.99 (intraclass correlation) for measurements of LA volume. In a sample of 10 randomly-selected patients, the interobserver intraclass correlation was 0.96 for measurements of EATtotal and 0.95 for measurements of the LA area (by N.G. and F.B.) The investigators were blinded in terms of AF status and blood sample measurements at the time of the CT measurements.

### Statistical Analysis

Differences in proportions were compared using a Chi-squared test or Fisher’s exact test as appropriate. Normally distributed continuous variables were expressed as mean ± SD and were compared using 2-sample t tests for independent samples. CRP, VEGF, IL-8, sICAM-1, TGF-β1, and vWF were expressed as median (25^th^–75^th^ percentile). These variables were log-transformed before being used in univariable and multivariable analyses. sICAM-1 was log-transformed despite its normal distribution to ensure internal consistency. Pearson correlations were performed to assess the associations between epicardial fat measurements and biomarkers.

Multivariable analyses were performed using multivariable linear analysis. Log-transformed blood levels of the biologic markers significantly associated with epicardial fat measurements on correlation analyses were used as dependent variables in these multivariable linear analyses. The epicardial fat measurements of interest were included as independent variables, as were BMI and LA volume. As the dependent variables were log-transformed, exponential transformations of β coefficients (Exp β) were also performed, which implies interpretation on a multiplicative scale. The level of biomarkers changes by 100*(Exp β-1)% for each one unit increase in the epicardial fat measurement of interest.

All tests were two-sided. A probability value less than 0.05 was considered significant. All statistical analyses were performed with SPSS 20 (SPSS Inc., Chicago, IL).

## Results

### Baseline Characteristics

The study population (n=49) was mainly comprised of middle-aged men (Table 1). Almost half of the population had hypertension, and the mean CHADS_2_ score was less than 1. The LA volume was much greater in patients with PeAF (*P*<0.001). Peripheral CRP was significantly lower in patients with PAF (median CRP 1.2 mg/L vs. 2.0 mg/L, *P*=0.04), but no significant differences were identified in the other biomarkers according to the type of AF regardless of the site of sampling.

**Table 1:** Characteristics and Levels of Biomarkers in the Peripheral and Left Atrial Blood in the Study Population According to the Type of Atrial Fibrillation.

	Study Population	PAF Group	PeAF Group	*P* Value
	(n=49)	(n=25)	(n=24)	(PAF vs. PeAF)
Age, years	59.3±8,5	60.0±9.0	58.5±8.1	0.55
Female gender, %	16.3%	20.0%	12.5%	0.70
BMI, kg/m^2^	27.6±4.2	26.4±3.4	28.8±4.6	0.05
BMI > 30 kg/m^2^, %	20.4%	16.0%	25.0%	0.50
Current smoker, %	8.2%	8.0%	8.3%	1.00
Hypertension, %	44.9%	40.0%	50.0%	0.48
Systolic blood pressure, mmHg	129.6±17.7	129.4±14.5	129.8±20.9	0.94
Diabetes mellitus, %	10.2%	12.0%	8.3%	0.67
Coronary artery disease, %	10.2%	12.0%	8.3%	1.00
History of cardiac failure, %	6.1%	4.0%	8.3%	0.53
History of stroke, %	8.2%	16.0%	0.0%	0.11
Chronic obstructive pulmonary disease or sleep apnea syndrome, %	14.3%	12.0%	16.7%	0.70
CHADS2 score	0.78±0.92	0.88±1.17	0.67±0.57	0.42
CHA2DS2-VASc score	1.4±1.3	1.56±1.53	1.25±1.11	0.42
LA volume - CT measurement, ml	97.0±30.2	77.8±18.5	116.9±27.0	<0,001
Biomarkers				
Peripheral CRP, μg/mL	1.8	1.2	2.0	0.04
	(0.8-3.7)	(0.5-3.7)	(1.2-4.4)	
Peripheral IL-8, pg/mL	7.0	7.0	7.0	0.17
	(4.3-9.0)	(2.0-8.5)	(5.0-10.0)	
LA IL-8, pg/mL	6.0	6.0	6.0	0.27
	(4.0-8.0)	(2.0-8.5)	(5.0-8.0)	
Peripheral sICAM-1, ng/mL	167.5	160.0	174.0	0.68
	(141.3-209.8)	(137.5-207.5)	(141.0-213.0)	
LA sICAM-1, ng/mL	164.0	155.0	167.0	0.66
	(133.5-207.0)	(132.0-203.5)	(142.0-207.0)	
Peripheral VWF, ng/mL	121.0	107.0	129.5	0.42
	(74.0-178.5)	(64.5-190.0)	(102.3-172.0)	
LA VWF, ng/mL	125.0	118.0	131.0	0.31
	(91.25-184.25)	(83.5-192.5)	(98.0-176.0)	
Peripheral VEGF, pg/mL	15.5	16.0	14.0	0.49
	(10.3-30.0)	(13.5-30.5)	(10.0-29.0)	
LA VEGF, pg/mL	11.0	14.0	11.0	0.35
	(7.0-16.8)	(8.5-20.5)	(4.0-15.0)	
Peripheral TGF-β1, ng/mL	11 981	11661	13809	0.94
	(8 939- 28 283)	(9 207-20 992)	(7 949-31 933)	
LA TGF- β1, ng/mL	11 572	11529	11614	0.97
	(7 949-18 980)	(7 410-21 354)	(8050-19 013)	

Abbreviations: BMI, body mass index; CRP, C-reactive protein; IL-8, interleukin-8; LA, left atrial; LVEF, left ventricular ejection fraction; PAF, paroxysmal atrial fibrillation; PeAF, persistent AF; sICAM-1, soluble intercellular adhesion molecule 1; TGF-β1, transforming growth factor-β1; VEGF, vascular endothelial growth factor; vWF, von Willebrand Factor.

Values shown are mean±SD, median (25th percentile-75th percentile), or n, %

Blood levels of biomarkers were compared using an unpaired t-test after log-transformation of the variables.

### EAT Volume and Regional Thickness of EAT in Patients with PAF and PeAF

Patients with PeF had higher EATatrial (*P*<0.05) and LA-ESO (*P*=0.04) and tended to have greater LA-ThA (*P*=0.06) compared to PAF patients (Table 2). In contrast, EATtotal and EATventricular, which were only slightly higher in patients with PeAF when compared to patients with PAF, were not significantly different between the two groups (*P*=0.18 for both). Other measures of regional fat were not significantly different between the two groups (*P*>0.20 for all).

**Table 2:** EAT Volumes and Regional EAT Thickness in the Study Population According to the Type of Atrial Fibrillation.

	Study Population	PAF Group	PeAF group	*P* Value
	(n=49)	(n=25)	(n=24)	(PAF vs. PeAF)
EATtotal, cm3	114.1±44.4	105.7±41.7	122.8±46.4	0.18
EATatrial, cm3	48.8±21.9	43.2±17.6	54.5±24.8	0.05
EATventricular, cm3	65.3±25.8	62.5±26.7	68.3±25.1	0.18
LA-ESO, mm	5.1±1.9	4.6±1.5	5.7±2.2	0.04
LA-ThA, mm	5.8±2.3	5.2±2.1	6.5±2.3	0.06
LA-PA, mm	6.0±2.9	6.5±3.0	5.5±2.7	0.25
IAS, mm	5.8±2.4	6.0±2.8	5.7±2.0	0.73
RAVG, mm	12.7±3.6	12.9±3.7	12.4±3.6	0.64
LAVG, mm	11.6±2.9	11.5±2.9	11.6±3.1	0.93
AIVG, mm	7.0±1.9	6.8±2.2	7.2±1.5	0.45

Abbreviations: AIVG, anterior interventricular groove; EAT, epicardial adipose tissue; EATatrial, atrial EAT; EATventricular, ventricular EAT; IAS, interatrial septum; LA-ESO, periatrial epicardial fat located between the mid left atrium and esophagus; LA-PA, periatrial epicardial fat located between mid left atrium and pulmonary artery; LA-ThA, periatrial epicardial fat located between the mid left atrium and thoracic aorta; LAVG, left atrioventricular groove; PAF, paroxysmal atrial fibrillation; PeAF, persistent AF; RAVG, right atrioventricular groove.

### Association Between EAT and Biomarkers

Univariable analysis showed that the levels of IL-8 and TGF-β1 were not associated with EAT measurements (Table 3). Only two significant correlations were found that involved VEGF. In contrast, LA-ESO, LA-ThA, LA-PA, IAS, and LAVG were significantly correlated with peripheral and LA vWF (*P*<0.01 for all except for LA-PA, which showed *P*<0.05). LA-ESO and LA-ThA were also significantly correlated with peripheral and LA sICAM-1 (*P*<0.02 for all). There were significant correlations between EAT volumes, LA-ESO, and LA-ThA.

**Table 3:** Correlations Between EAT Variables and Biomarkers.

	Peripheral		Peripheral		LA		Peripheral		LA		Peripheral		LA		Peripheral		LA		Peripheral		LA	
	CRP		IL-8		IL-8		sICAM-1		sICAM-1		vWF		vWF		VEGF		VEGF		TGF-β1		TGF-β1	
	Corr	*P* value	Corr	*P* value	Corr	*P* value	Corr	*P* value	Corr	*P* value	Corr	*P* value	Corr	*P* value	Corr	*P* value	Corr	*P* value	Corr	*P* value	Corr	*P* value
BMI	**0.397**	**0.008**	0.097	0.51	0.115	0.44	0.221	0.13	0.268	0.07	0.097	0.51	0.033	0.82	0.006	0.97	0.002	0.99	0.096	0.52	0.12	0.42
EATtotal /10 cm3	0.273	0.08	0.097	0.51	0.025	0.87	0.241	0.10	0.233	0.11	0.087	0.55	0.011	0.94	0.076	0.61	-0.162	0.27	0.035	0.81	0.006	0.97
EATatrial /10 cm3	**0.301**	**0.05**	0.081	0.58	0.04	0.79	0.198	0.18	0.215	0.14	0.124	0.40	0.105	0.48	0.064	0.67	-0.166	0.26	0.04	0.79	0.006	0.97
EATventricular /10 cm3	0.211	0.17	0.097	0.51	0.01	0.95	0.246	0.09	0.219	0.14	0.044	0.76	-0.071	0.63	0.077	0.60	-0.138	0.35	0.027	0.86	0.005	0.97
LA-ESO /mm	**0.356**	**0.02**	-0.068	0.64	-0.002	0.99	**0.381**	**0.008**	**0.337**	**0.019**	**0.440**	**0.002**	**0.432**	**0.002**	0.107	0.47	0.059	0.69	0.000	1	0.073	0.62
LA-ThA /mm	**0.361**	**0.02**	-0.13	0.39	0.057	0.71	**0.441**	**0.002**	**0.387**	**0.008**	**0.399**	**0.005**	**0.503**	**<0.001**	0.194	0.20	0.326	**0.03**	-0.066	0.66	0.111	0.46
LA-PA /mm	0.034	0.83	0.084	0.57	0.132	0.37	-0.081	0.58	-0.195	0.18	**0.310**	**0.03**	**0.335**	**0.02**	0.369	**0.01**	0.119	0.42	0.221	0.13	0.228	0.12
IAS /mm	0.062	0.69	-0.064	0.667	0.072	0.626	0.009	0.951	0.011	0.94	**0.408**	**0.004**	**0.527**	**<0.001**	0.162	0.27	0.045	0.762	0.028	0.853	0.089	0.55
RAVG /mm	0.059	0.71	0.053	0.723	0.127	0.391	0.12	0.415	0.126	0.393	**0.298**	**0.038**	0.200	0.174	-0.017	0.907	0.192	0.192	0.046	0.758	0.098	0.506
LAVG /mm	0.281	0.07	-0.012	0.937	0.034	0.816	0.247	0.091	0.269	0.065	**0.449**	**0.001**	**0.457**	**0.001**	0.122	0.408	0.094	0.526	0.115	0.438	0.07	0.638
AIVG /mm	0.154	0.33	0.236	0.107	0.153	0.299	0.244	0.095	0.233	0.111	0.100	0.493	0.054	0.714	0.114	0.44	-0.005	0.971	0.128	0.385	0.153	0.298

Pearson correlations were used after log-transformation of the biomarker variables.

Abbreviations: AIVG, anterior interventricular groove; Corr, correlations; EAT, epicardial adipose tissue; EATatrial, atrial EAT; EATventricular, ventricular EAT; IAS, interatrial septum; LA-ESO, periatrial epicardial fat located between the mid left atrium and esophagus; LA-PA, periatrial epicardial fat located between mid left atrium and pulmonary artery; LA-ThA, periatrial epicardial fat located between the mid left atrium and thoracic aorta; LAVG, left atrioventricular groove; PAF, paroxysmal atrial fibrillation; PeAF, persistent AF; RAVG, right atrioventricular groove.

Except for the LA volume, which was significantly associated with peripheral sICAM-1 (r=0.300, *P*=0.04), and BMI, which was significantly associated with LA sICAM1 (r=0.397, *P*=0.008), the other baseline characteristics were not significantly associated with CRP, sICAM1, and vWF levels.

Multivariable linear regression models were then constructed that were adjusted for BMI and LA volume (Tables 4 and 5). In multivariable analysis, EATatrial and EAT regional thickness variables were not significantly associated with hsCRP. In contrast, higher LA-ESO and LA-ThA were both independently associated with higher peripheral sICAM-1 (5.2% increase in sICAM-1 blood levels for each 1-mm increase in LA-ESO or LA-ThA, *P*=0.04 and *P*=0.01, respectively). Only LA-ThA was independently associated with LA sICAM-1. LA-ESO, LA-ThA, LA-PA, IAS, and LAVG were all independently associated with both peripheral and LA sICAM-1 (increase in sICAM-1 ranging from 6.3% to 12.8% for a 1-mm increase in the EAT thickness variable depending on the considered measurement).

**Table 4:** Multivariable Models with Peripheral and LA Blood Levels of sICAM-1 as the Dependent Variable.

	Study population				PAF group				PeAF group			
	Peripheral		LA		Peripheral		LA		Peripheral		LA	
	sICAM-1		sICAM-1		sICAM-1		sICAM-1		sICAM-1		sICAM-1	
	Exp β	*P* value	Exp β	*P* value	Exp β	*P* value	Exp β	*P* value	Exp β	*P* value	Exp β	*P* value
EATtotal /10 cm3	1.010	0.34	1.008	0.47	0.999	0.97	1.003	0.88	1.018	0.09	1.013	0.24
EATatrial /10 cm3	1.016	0.48	1.016	0.49	0.983	0.72	0.994	0.91	1.038	0.07	1.032	0.13
EATventricular /10 cm3	1.019	0.32	1.012	0.52	1.008	0.84	1.012	0.75	1.026	0.18	1.014	0.50
LA-ESO /mm	**1.052**	**0.04**	1.042	0.10	**1.114**	**0.05**	1.103	0.09	1.036	0.11	1.026	0.27
LA-ThA /mm	**1.052**	**0.01**	**1.042**	**0.04**	**1.083**	**0.02**	**1.080**	**0.02**	1.030	0.23	1.012	0.64
LA-PA /mm	0.993	0.64	0.978	0.14	0.964	0.13	**0.950**	**0.04**	1.029	0.10	1.012	0.51
IAS /mm	0.999	0.95	0.999	0.98	0.993	0.80	0.999	0.97	1.011	0.65	1.000	0.98
RAVG /mm	1.003	0.80	1.004	0.74	0.988	0.53	0.992	0.69	1.015	0.31	1.013	0.39
LAVG /mm	1.018	0.23	1.021	0.17	1.012	0.64	1.027	0.32	1.022	0.17	1.015	0.37
AIVG /mm	1.030	0.18	1.027	0.23	1.034	0.31	1.029	0.39	1.044	0.17	1.042	0.20

Models were adjusted for BMI and LA volume. As the dependent variable was log-transformed, the β coefficients were exponentially transformed. Exponential transformation of β coefficients (Exp β) implies an interpretation on a multiplicative scale. The level of sICAM1 changes by 100*(Exp β-1) % for a one unit increase in each EAT variable.

Abbreviations: AIVG, anterior interventricular groove; EAT, epicardial adipose tissue; EATatrial, atrial EAT; EATventricular, ventricular EAT; IAS, interatrial septum; LA-ESO, periatrial epicardial fat located between the mid left atrium and esophagus; LA-PA, periatrial epicardial fat located between mid left atrium and pulmonary artery; LA-ThA, periatrial epicardial fat located between the mid left atrium and thoracic aorta; LAVG, left atrioventricular groove; PAF, paroxysmal atrial fibrillation; PeAF, persistent AF; RAVG, right atrioventricular groove.

**Table 5:** Multivariable Models with Peripheral and LA Blood Levels of vWF as the Dependent Variable.

	Study population				PAF group				PeAF group			
	Peripheral		LA		Peripheral		LA		Peripheral		LA	
	vWF		vWF		vWF		vWF		vWF		vWF	
	Exp β	*P* value	Exp β	*P* value	Exp β	*P* value	Exp β	*P* value	Exp β	*P* value	Exp β	*P* value
EATtotal /10 cm3	1.005	0.78	0.999	0.97	0.977	0.53	0.956	0.21	1.0178	0.44	1.024	0.23
EATatrial /10 cm3	1.023	0.55	1.025	0.49	0.923	0.31	0.901	0.16	1.060	0.17	**1.077**	**0.04**
EATventricular /10 cm3	0.999	0.96	0.980	0.52	0.985	0.81	0.947	0.34	1.00	0.92	1.011	0.77
LA-ESO /mm	**1.128**	**0.002**	**1.123**	**0.002**	1.172	0.09	1.122	0.20	**1.110**	**0.02**	**1.124**	**0.001**
LA-ThA /mm	**1.092**	**0.008**	**1.114**	**<0.001**	1.113	0.07	**1.121**	**0.048**	1.076	0.12	**1.139**	**0.001**
LA-PA /mm	**1.063**	**0.02**	**1.064**	**0.01**	**1.092**	**0.03**	1.053	0.19	1.039	0.33	**1.096**	**0.002**
IAS /mm	**1.093**	**0.003**	**1.112**	**<0.001**	**1.122**	**0.003**	**1.105**	**0.008**	1.033	0.54	**1.131**	**0.002**
RAVG /mm	1.042	0.05	1.028	0.18	1.046	0.16	1.015	0.64	1.047	0.14	**1.057**	**0.03**
LAVG /mm	**1.083**	**0.001**	**1.080**	**0.001**	**1.108**	**0.01**	**1.094**	**0.02**	1.058	0.09	**1.066**	**0.02**
AIVG /mm	1.027	0.51	1.013	0.72	1.035	0.53	1.013	0.80	1	1	0.997	0.96

Models were adjusted for BMI and LA volume. As the dependent variable was log-transformed, the β coefficients were exponentially transformed. Exponential transformation of β coefficients (Exp β) implies an interpretation on a multiplicative scale. The level of vWF changes by 100*(Exp β-1) % for a one unit increase in each EAT variable.

Abbreviations: AIVG, anterior interventricular groove; EAT, epicardial adipose tissue; EATatrial, atrial EAT; EATventricular, ventricular EAT; IAS, interatrial septum; LA-ESO, periatrial epicardial fat located between the mid left atrium and esophagus; LA-PA, periatrial epicardial fat located between mid left atrium and pulmonary artery; LA-ThA, periatrial epicardial fat located between the mid left atrium and thoracic aorta; LAVG, left atrioventricular groove; PAF, paroxysmal atrial fibrillation; PeAF, persistent AF; RAVG, right atrioventricular groove.

In the subset of patients with PAF, greater LA-ESO and LA-ThA were significantly associated with higher peripheral sICAM-1 (P<0.05) and tended to be associated with higher peripheral vWF (P=0.09 and P=0.07 respectivelly. LA-ThA was also significantly associated with LA sICAM-1 and LA vWF (Table 4 and 5). In the subset of patients with PeAF, all periatrial EAT thickness variables were independently associated with LA vWF. Greater EATatrial also tended to be associated with higher LA vWF (Table 4 and 5).

## Discussion

We found that the presence of greater amounts of EAT in contact with the LA (mainly LA-ESO and LA-ThA) was significantly associated with increased levels of sICAM-1 and vWF in patients with AF. These results suggest that periatrial EAT, possibly through a paracrine mechanism, may modulate endothelial dysfunction in patients with AF. The presence of abnormal endothelium could in turn underlie the well-described association between epicardial fat and poorer outcome of AF patients.

In the present study, the significant association between periatrial EAT and CRP observed in the univariable analyses became insignificant when adjusting for BMI and LA volume. The other inflammatory biomarkers, i.e. IL-8, and TGF-β1, were not significantly correlated with EAT variables. To some extent, these negative results contradict the recent hypothesis that there is a link between EAT and AF [4,13,26]. However, we only measured LA blood levels of inflammatory markers. As the EAT and the LA only weigh a few grams, an inflammatory process localized within the EAT and the LA is likely incapable of generating higher LA blood levels of pro-inflammatory cytokines. The production of inflammatory cytokines by human EAT has been elegantly shown using EAT biopsies [27].

We found a strong independent association between periatrial EAT and markers of endothelial dysfunction as measured by vWF and sICAM1. sICAM1 is an adhesion molecule triggered by inflammation that has a key role in the genesis of endothelial dysfunction [28–30]. EATventricular, EATtotal, and AIVG were not significantly associated with markers of endothelial dysfunction, even in the univariable analysis. This suggests that the EAT surrounding the LA, rather than the EAT itself, is a key determinant of endothelial dysfunction in AF patients. 

Our findings suggest that the local inflammation generated by periatrial EAT triggers substantial endothelial dysfunction within the LA, which, in turn translates into higher systemic markers of endothelial dysfunction. Translational evidence supports a causal impact of visceral fat on the genesis of endothelial dysfunction the activation of the NF-κB pathway [15]. Only one study has reported that abnormal EAT is associated with endothelial dysfunction [18]. That study focused on sinus rhythm patients and measured EAT using echocardiography; this precludeds specific investigation of the impact of atrial EAT vs. ventricular EAT. To the best of our knowledge, our study is the first to report the considerable impact of periatrial EAT on the level of markers of endothelial dysfunction in patients with AF. Considering that endothelial dysfunction, as measured by vWF blood levels, is strongly associated with AF-related strokes[31,32], this result is clinically relevant. Because it is a strong predictor of higher vWF blood levels, higher periatrial EAT might also be associated with higher stroke incidence in patients with AF. 

In the present study, only the posterior LA fat thickness was associated with sICAM1 blood levels. In the study published by Batal and colleagues, only the LA-ESO was independently associated with the type of AF (PAF vs. PeAF) [4]. In our dataset, only atrial EAT and LA-ESO were significantly increased in patients with PeAF compared to patients with PAF. Thus, posterior LA fat thickness is closely related to the persistence of AF and endothelial disturbance in patients with AF. The observation that posterior LA fat thickness is a better marker of endothelial dysfunction than the other EAT variables might be explained by neural considerations. Ganglionated plexuses are located in the epicardial fat pads, mainly next to the posterior wall of the LA [33]. These plexuses are important for the maintenance of AF [12] and could be influenced by the inflammation of neighboring EAT [13]. Consequently, the EAT localized next to the posterior wall of the LA might have a large impact on atrial electrophysiology and endothelial function [34].

Importantly, we did not find significant associations of markers of endothelial dysfunction with regional adiposity (i.e. EATventricular and AIVG) or total adiposity as measured by BMI. These lacks of association with adiposity measurement that do not focus on local (i.e. periatrial) fat underline the importance of fat location rather than abundance.

### Limitations

This was a single center study with limited statistical power. However, despite the limited sample size in our study, we identified significant associations between periatrial EAT and both sICAM-1 and vWF levels that were independent of BMI and LA volume. Larger multicenter studies are needed to quantify the association between the different EAT measurements and markers of inflammation/endothelial dysfunction. Furthermore, only patients who underwent AF ablation were included in this study. These patients do not represent all patients with AF; in particular, they may not be representative of patients with PeAF. Patients with long-lasting AF are rarely treated with AF ablation. Additional studies should be conducted that include an analysis of peripheral blood samples of inflammation and endothelial dysfunction biomarkers.

### Implications

Our study suggests that the thickness of the posterior EAT localized between the LA and the esophagus (LA-ESO) or thoracic aorta (LA-ThA), which can easily be measured using cardiac CT, is associated with increased levels of biomarkers of endothelial dysfunction in the peripheral blood and LA independently of BMI and LA volume. By focusing on the link between epicardial fat and AF, these findings may advance our understanding of AF pathogenesis. From a clinical standpoint, measurement of LA-ESO and LA-ThA thickness during cardiac CT, which is routinely performed before AF ablation, may be important for assessing the level of endothelial dysfunction. Further studies are needed to determine whether this assessment of endothelial dysfunction in patients with AF translates into a better risk assessment of stroke.

## Conclusions

We found that the presence of more periatrial EAT was significantly associated with increased levels of biomarkers of endothelial dysfunction in patients with AF. No such associations were identified with BMI. These findings suggest that periatrial EAT may modulate endothelial dysfunction, possibly through a paracrine mechanism, and that this modulation might in turn influence the prognosis of AF patients. These results suggest a potential role for pericardial EAT quantification to risk-stratify this growing patient population.
